# Total knee arthroplasty in patients with degenerative spine disease: does spinal fusion affect outcomes? A matched comparative analysis using a national database

**DOI:** 10.1186/s43019-025-00267-4

**Published:** 2025-03-24

**Authors:** Mohammad Daher, Jonathan Liu, Alan H. Daniels, Eric M. Cohen, Valentin Antoci, Mouhanad M. El-Othmani

**Affiliations:** Department of Orthopedics, Brown University, 1 Kettle Point Avenue, Providence, RI 02914 USA

**Keywords:** Total knee arthroplasty, Knee osteoarthritis, Degenerative spine disease, Spinal fusion

## Abstract

**Background:**

The need for total knee arthroplasty (TKA) and spinal fusion (SF) for degenerative spine disease (DSD) is increasing. However, it is still unknown if prior SF for DSD impacts outcomes following TKA. This study aims to fill this gap by comparing the risk of complications and revisions in patients undergoing TKA with DSD between patients with and without SF.

**Methods:**

This study is a retrospective review of the PearlDiver Mariner Database between 2010 and 2020. On the basis of whether or not patients had had prior SF, the patients undergoing TKA were divided into two groups: patients with DSD and SF and patients with DSD and without SF. The two groups were matched on the basis of age, gender, the Charlson Comorbidity Index (CCI), and obesity. Surgical complications (mechanical loosening, prosthetic dislocation, periprosthetic fractures, and stiffness) and revisions at 1, 2, and 3 years were compared between the groups.

**Results:**

The patients in the TKA with DSD and no SF cohort were older (64.9 ± 8.4 versus 63.3 ± 8.1 years, *p* < .001), had higher CCI (2.0 ± 2.2 versus 1.6 ± 2.0, *p* < .001), and had a lower rate of obesity (58.7% versus 61.7%, *p* < .001). After being matched, 8887 patients remained in each group. There was a higher rate of stiffness and manipulation under anesthesia (MUA) in the no-fusion cohort at 1 year (0.7% versus 0.1%, *p* < .001; and 0.5% versus 0.2%, *p* < .001, respectively), 2 years (1.2% versus 0.5%, *p* < .001; and 1.1% versus 0.6%, *p* < .001, respectively), and 3 years (1.7% versus 0.7%, *p* < .001; and 1.6% versus 0.9%, *p* < .001, respectively).

**Conclusions:**

This study shows no increase in risk of surgical complications and revisions after TKA in patients with DSD and SF compared with patients without SF. Notably, SF was shown to be protective of stiffness and MUA after TKA in patients with DSD.

## Introduction

Globally, the prevalence of osteoarthritis (OA) increased by 113.25% between 1990 and 2019, with the knee joint being the most affected area [1–3]. For advanced and end-stage OA of the knee joint, total knee arthroplasty (TKA) is advised as a successful therapeutic option to address pain and functional limitations when conservative approaches are unable to provide symptomatic relief [4]. In fact, TKA was the second-fastest growing surgery in the USA between 2003 and 2012, going from 421,700 to 700,100 procedures annually, and this trend is projected to continue [5].

Similarly, the prevalence of degenerative spinal diseases (DSD) has been steadily rising owing to the aging population and longer life expectancies [6, 7]. In fact, DSD are among the most prevalent causes of chronic low back pain and disability worldwide [8]. These conditions include degenerative disc disease, spondylolysis, and spondylolisthesis [8]. They result from progressive structural and functional changes in the spine that lead to nerve impingement, instability, and deformity developing at specific spinal levels [9]. DSD can be surgically managed with spinal fusion (SF), which can correct the lumbar deformity, improve neurological function, and restore segmental lordosis, which has potential to greatly enhanced patient outcomes. [10–15] In fact, spinal conditions were previously shown to impact TKA outcomes, with preexisting deformities leading to lower patient-reported outcome measures (PROMs) and poorer operative outcomes including decreased range of motion and increased incidence of knee flexion contracture [16–19]. However, to date, evidence on outcomes of TKA among patients with DSD with or without SF remains lacking in literature.

As such, the aim of this study is to assess the surgical complications and rates of revision after TKA among patients with a previous diagnosis of DSD and compare them between those who underwent SF prior to the TKA and those who did not. We hypothesized that that there would be no increase in risk of surgical complications and revisions after TKA in patients with DSD and SF compared with patients without SF.

## Material and methods

### Study design

The PearlDiver Mariner Database (PearlDiver Technologies Inc., Colorado Springs, CO, USA) was reviewed retrospectively for this investigation. Health Insurance Portability and Accountability Act (HIPAA) regulations are followed in the format in which the deidentified patient record data are received. The data includes all payer categories with a medical claim. The database contains codes from the ninth and tenth editions of the International Classification of Diseases (ICD), as well as current procedural technology (CPT) codes for hospital and physician billing records, and outpatient filled prescription records. Institutional review board clearance was not required for this study given the deidentified nature of included data in the PearlDiver database.

### Cohorts

Patients were included if they underwent primary elective TKA between 2010 and 2020 with a preexisting diagnosis of lumbar spinal stenosis, as identified through ICD-9 and ICD-10 codes. To ensure adequate follow-up, only patients with at least 3 years of postoperative data were included.

**Fig. 1:**
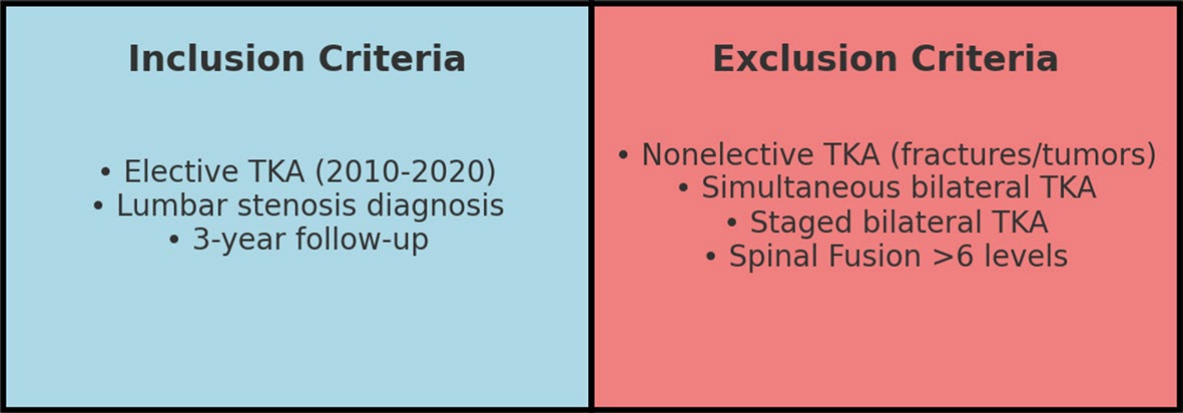
Inclusion and exclusion criteria

Exclusion criteria consisted of patients undergoing TKA for nonelective reasons, such as fractures or tumors/metastases, as well as those undergoing simultaneous or staged bilateral TKA. Patients who had undergone SF involving more than six levels were also excluded to maintain homogeneity in the SF group (Fig. 1).

Patients were then separated into two groups on the basis of whether or not they had SF of a maximum of six levels prior to the TKA. The two groups were then matched by age, gender, obesity, and the Charlson Comorbidity Index (CCI). The CCI, developed in 1987, is the most widely used index and is often considered to be the gold-standard measure to assess comorbidity in clinical research. It consists of 19 items corresponding to different medical comorbid conditions displaying different clinical weights on the basis of the adjusted risk of 1-year mortality [20, 21]. A higher CCI indicates that the patient has multiple or severe comorbidities, leading to a higher risk of complications, mortality, and poorer health outcomes. In contrast, a lower CCI suggests fewer or less severe comorbidities, meaning a lower risk of complications and better overall health outcomes.

### Data collection

The collected data included patient demographics (age, gender), baseline CCI, and surgical outcomes at 1, 2, and 3 years postoperatively. The primary outcomes assessed were surgical complications including:

- Mechanical loosening
- Prosthetic dislocation
- Periprosthetic fractures
- Postoperative stiffness

Additionally, rates of revision surgery, lysis of adhesions (LOA), and manipulation under anesthesia (MUA) were recorded at each follow-up interval.

### Statistical analysis

Statistical analysis was performed using R 4.0.3 (R Foundation for Statistical Computing, Vienna, Austria, https://www.R-project.org/). Continuous variables were presented using means and standard deviations (SD) and compared between the two groups using the independent samples Student *t*-test. Categorical variables were presented as frequencies and percentages, and comparison between the two groups was performed using the chi-squared test. The cutoff point for statistical significance was established as *p* < 0.05 (Table 1).

**Table 1:** Demographic and baseline characteristics of the matched and nonmatched cohorts

Variables	Not matched			Matched		
	TKA with no SF	TKA after SF	*p*-Value	TKA with no SF	TKA after SF	*p*-Value
*n*	199,273	8906	–	8887	8887	–
Age, years (mean ± SD)	64.9 ± 8.4	63.3 ± 8.1	< .001	63.3 ± 8.1	63.3 ± 8.1	1
Gender (male/female)	73,185/126,088	3335/5571	0.17	3321/5566	3321/5566	1
Charlson Comorbidity Index	2.0 ± 2.2	1.6 ± 2.0	< .001	1.6 ± 2.0	1.6 ± 2.0	1
Obesity (*n* (%))	116,954 (58.7%)	5492 (61.7%)	< .001	5483 (61.7%)	5483 (61.7%)	1

## Results

### Demographics and baseline characteristics

**Fig. 2:**
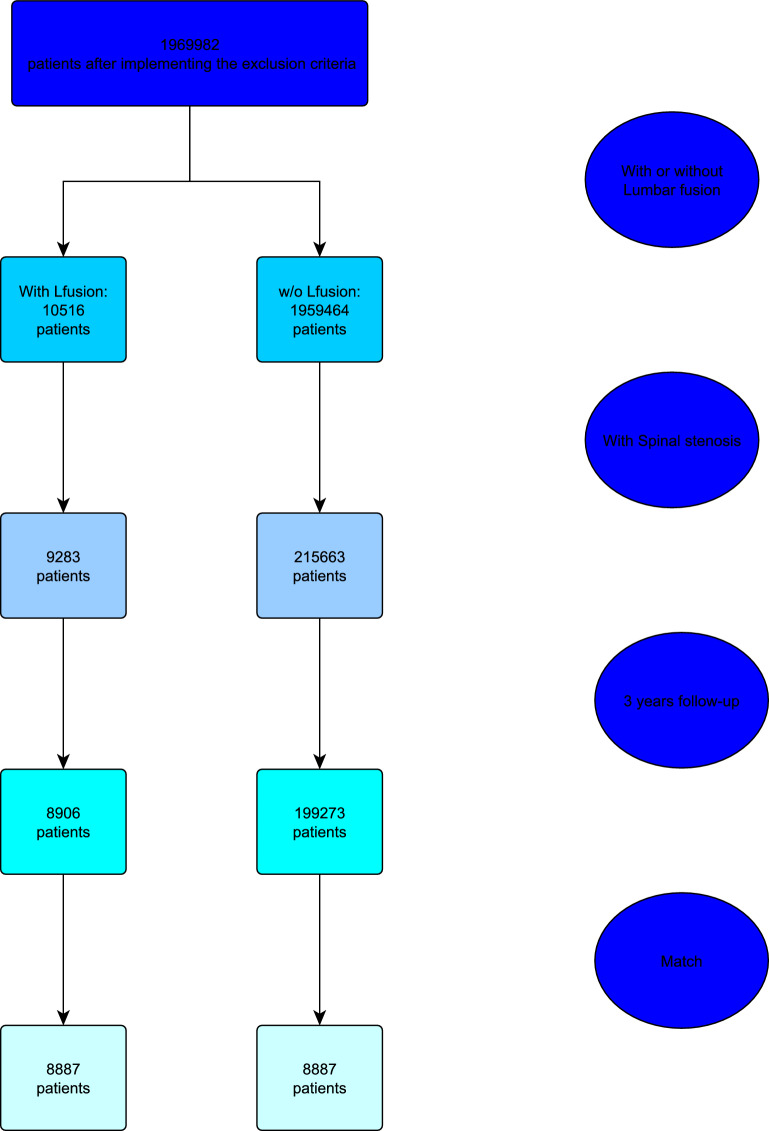
Flow diagram of patients’ inclusion

There were 199,273 patients in the spinal stenosis with no SF TKA group and 8906 patients in the SF TKA group (Fig. 2). The patients in the TKA with no SF cohort were older (64.9 ± 8.4 versus 63.3 ± 8.1 years, *p* < 0.001), had higher CCI (2.0 ± 2.2 versus 1.6 ± 2.0, *p* < 0.001), and had a lower incidence of obesity overall (58.7 versus 61.7%, *p* < 0.001). There was no difference in the proportion of males between the two unmatched cohorts (36.7 versus 37.4%, *p* = 0.17). After matching, 8887 patients were retained in each group, with a mean age of 63.3 ± 8.1 years, a mean CCI of 1.6 ± 2.0, 37.3% males, and 61.7% with obesity (Table 1).

### Surgical complications and revisions

There was a higher rate of stiffness and MUA in the no fusion group at 1 year (0.7 versus 0.1%, *p* < 0.001; and 0.5 versus 0.2%, *p* < 0.001, respectively), 2 years (1.2 versus 0.5%, *p* < 0.001; and 1.1 versus 0.6%, *p* < 0.001, respectively), and 3 years (1.7 versus 0.7%, *p* < 0.001; and 1.6 versus 0.9%, *p* < 0.001, respectively). However, at 1 year, there was no difference between the two groups in mechanical loosening (0.3 versus 0.4%, *p* = 0.21), prosthetic dislocation (0.2% versus 0.2%, *p* = 1.00), periprosthetic fractures (*p* = 1.00), LOA (< 10 versus < 10, *p* = 0.68), or the number of revision surgeries (0.2 versus 0.4%, *p* = 0.17). Similar results were seen at 2 years and 3 years for mechanical loosening (0.6 versus 0.9%, *p* = 0.08; and 0.9 versus 1.2%, *p* = 0.06, respectively), prosthetic dislocations (0.4 versus 0.4%, *p* = 0.81; and 0.7% versus 0.6%, *p* = 0.41, respectively), periprosthetic fractures (0.1 versus 0.1%, *p* = 1.00; and 0.3 versus 0.2%, *p* = 1.00, respectively), LOA (0.1% versus < 10, *p* = 0.17; and 0.2 versus 0.1%, *p* = 0.57, respectively), and revision surgeries (0.7 versus 0.9%, *p* = 0.23; and 1.2 versus 1.3%, *p* = 0.54, respectively) (Table 2).

**Table 2:** Surgical complications in TKA with and without spinal fusion. TKA, total knee arthroplasty; SF, spinal fusion

Variables	Timepoint	TKA with no SF	TKA after SF	*p*-Value
Mechanical loosening, *n* (%)	1 year	27 (0.3%)	38 (0.4%)	0.21
	2 years	55 (0.6%)	76 (0.9%)	0.08
	3 years	80 (0.9%)	107 (1.2%)	0.06
Prosthetic dislocation, *n* (%)	1 year	15 (0.2%)	14 (0.2%)	1.00
	2 years	37 (0.4%)	34 (0.4%)	0.81
	3 years	65 (0.7%)	55 (0.6%)	0.41
Periprosthetic fractures, *n* (%)	1 year	< 10	< 10	1.00
	2 years	11 (0.1%)	11 (0.1%)	1.00
	3 years	23 (0.3%)	22 (0.2%)	1.00
Stiffness, *n* (%)	1 year	59 (0.7%)	12 (0.1%)	< 0.001
	2 years	109 (1.2%)	44 (0.5%)	< 0.001
	3 years	147 (1.7%)	65 (0.7%)	< 0.001
Lysis of adhesions (%)	1 year	< 10	< 10	0.68
	2 years	13 (0.1%)	< 10	0.17
	3 years	16 (0.2%)	12 (0.1%)	0.57
Manipulation under anesthesia, *n* (%)	1 year	48 (0.5%)	17 (0.2%)	< 0.001
	2 years	98 (1.1%)	54 (0.6%)	< 0.001
	3 years	141 (1.6%)	83 (0.9%)	< 0.001
Revision, *n* (%)	1 year	21 (0.2%)	32 (0.4%)	0.17
	2 years	61 (0.7%)	76 (0.9%)	0.23
	3 years	104 (1.2%)	114 (1.3%)	0.54

## Discussion

With the prevalence of both DSD and knee OA increasing owing to aging populations, surgeons will increasingly encounter patients undergoing TKA with coexisting spinal pathology, with or without prior SF. Despite the biomechanical interplay between spinal alignment and lower extremity function, the impact of SF on TKA outcomes remains largely unexplored. This gap in literature limits surgeons’ ability to provide patients with data-driven counseling regarding potential postoperative risks and expected functional outcomes. Our findings highlight key baseline differences between patients with and without prior SF. Notably, patients without SF were significantly older and had higher comorbidity burdens. After matching the two cohorts, we observed a higher incidence of post-TKA stiffness and the need for MUA in patients without SF. However, importantly, no significant differences were observed in mechanical loosening, prosthetic dislocation, periprosthetic fractures, lysis of adhesions LOA, or revision rates between the two groups over a 3-year follow-up period.

The results of this study highlight older and increasing medical comorbidities among patients that did not undergo SF compared with those who did. This finding could potentially be explained by the increasing risk of perioperative complications among this population, prohibiting them from undergoing complex extensive spinal procedures. While this patient population could be considered as unlikely surgical candidate for major spinal procedures, they successfully underwent TKA without added major complications. In a previous study comparing outcomes between total joint arthroplasty and spinal fusion, patients undergoing TKA were older than patients undergoing SF [22]. This finding was reported in several studies [23–25]. These observations could be explained by the fact that in most cases, TKA is a relatively safer procedure that does not require a long operative time generally, and it can be done safely in the senior population [26–28].

Studies have explored the impact of SF on the surgical outcomes of THA. A recent meta-analysis reported a higher rate of periprosthetic fracture, prosthetic dislocation, overall complications, and revisions after THA in patients with a prior SF compared with patients undergoing THA without SF [29]. Regarding TKA, our study showed that patients with SF had a lower incidence of stiffness and MUA after TKA. Knee stiffness after TKA can result from various causes, with adhesions (arthrofibrosis) being the most common. Surgical complications, such as fractures, infections, or wound healing issues, may require periods of immobilization, further contributing to stiffness. Rarely, incorrect positioning or sizing of the implant during surgery can also cause persistent stiffness that may require a revision surgery. Management often involves MUA, a noninvasive procedure aimed at improving motion in stiff knees [30, 31]. Our findings showing a lower incidence of stiffness and MUA after TKA in patients with SF could be potentially explained by the reduced extent of compensation needed such as knee flexion in patients with DSD after SF. In fact, managing the spine first would result in proper alignment of the spine, leading to a resolution of the compensatory mechanisms. However, SF did not have an influence on the remaining surgical complications or revisions after TKA in patients with DSD. While this was not previously explored in literature, some studies explored the impact of spinal disease on patient reported outcome measures (PROMs) and spinopelvic alignment after TKA. Ayers et al. assessed the relationship between back pain intensity and patient satisfaction after TKA, finding that severe back pain was significantly associated with increased dissatisfaction 1 year after TKA [32]. In addition, Shichman et al. assessed changes in spinopelvic alignment following TKA, highlighting that patients with prior SF compared with patients without SF experienced significant alterations in pelvic tilt and sacral slope [33]. In contrast, a recent study by Daher et al. showed that TKA did not have an impact on the spinopelvic alignment or PROMs of patients with adult spinal deformity [34].

These results provide valuable clinical insight, indicating that TKA outcomes, in terms of long-term implant stability and revision risk, are not adversely affected by prior spinal fusion. While increased post-TKA stiffness and MUA rates in the non-SF group warrant further investigation, the lack of differences in major complications and revision rates suggests that SF does not impose additional risk in patients undergoing TKA. This evidence may assist orthopedic and spine surgeons in shared decision-making, helping them counsel patients on the expected risks and benefits of undergoing TKA with or without a history of SF. In addition, the results of this study lead us to advocate for a spinal fusion prior to TKA in case of operable spinal stenosis, when properly indicated. However, future single-center or multicenter studies are needed to confirm our findings and further assess the impact of SF on patient-reported outcome measures of TKA compared with patients with DSD and with no SF. Nevertheless, patients indicated to undergo both SF and TKA should be informed about the findings in this study to be able to make an informed decision regarding the order of their surgeries.

There are certain limitations with this study. First, this study is a retrospective comparative analysis; as such, its results are subject to the biases inherent to its nature. Second, the study’s findings depend on precise coding in the used database [35]. Third, while we selected patients that had DSD before having SF, their spinal procedure could have been done for adult spinal deformity or other indications, even though the SF procedure we selected had a maximum of six levels of fusion. Last, other confounding factors not accounted for could be contributing to the difference in stiffness and MUA seen between the two groups. Future prospective studies are required to have access to more granular data regarding the indications of the spinal procedure and confirm our findings.

## Conclusions

This study shows no increase in risk of surgical complications and revisions after TKA in patients with DSD and SF compared with patients without SF. Notably, SF was shown to be protective of stiffness and MUA after TKA in patients with DSD.

## Data Availability

Data are available upon reasonable request from the corresponding author.

## Abbreviations

TKA: Total knee arthroplasty
SF: Spinal fusion
DSD: Degenerative spine disease
CCI: Charlson Comorbidity Index
MUA: Manipulation under anesthesia
PROMs: Patient-reported outcome measures
ICD: International Classification of Diseases
CPT: Current procedural technology
LOA: Lysis of adhesions
